# Management of Blunt Thoracic Aortic Injury in Polytrauma Patients: A Review of Clinical Outcomes and Intervention Strategies

**DOI:** 10.7759/cureus.82400

**Published:** 2025-04-16

**Authors:** Aliaa H Alkhazendar, Manahil Awan, Mawada Taha, Shahzad Ahmad, Syed Muhammad Baqar Raza, Deewan Aakash

**Affiliations:** 1 Surgery, The Islamic University of Gaza, Gaza, PSE; 2 General Medicine, Liaquat National Hospital, Karachi, PAK; 3 General Surgery, The National Ribat University, Khartoum, SDN; 4 Surgery, Liaquat National Hospital, Karachi, PAK; 5 Cardiac Surgery, Rawalpindi Institute of Cardiology, Rawalpindi, PAK; 6 Vascular Surgery, Evangelisches Krankenhaus Hubertus, Berlin, DEU

**Keywords:** aortic rupture, blunt thoracic aortic injury, endovascular repair, intervention timing, polytrauma, systematic review, tevar, trauma surgery, vascular trauma

## Abstract

Blunt thoracic aortic injury (BTAI) is a life-threatening condition most commonly resulting from high-energy trauma, such as motor vehicle accidents. It is frequently associated with polytrauma, which complicates clinical decision-making and the timing of surgical intervention. This systematic review aimed to evaluate the effectiveness, safety, and outcomes of different management strategies, particularly thoracic endovascular aortic repair (TEVAR), in patients with BTAI and concurrent polytrauma. A comprehensive search was conducted across five major databases, PubMed, Embase, Scopus, Web of Science, and the Cochrane Library per PRISMA guidelines, identifying 378 records. Five studies were included after screening and eligibility assessment, comprising clinical trials, cohort studies, and narrative reviews. The results consistently favored TEVAR over open repair, demonstrating high technical success, lower perioperative morbidity, and reduced mortality, especially when intervention was appropriately timed. The review also highlighted the influence of injury severity, hemodynamic stability, and technological advancements on treatment selection and outcomes. While TEVAR has emerged as the preferred approach in modern trauma care, gaps in comparative long-term data and standardized protocols persist. Further prospective multicenter studies are recommended to strengthen the evidence base and refine clinical guidelines.

## Introduction and background

Blunt aortic injury (BAI) remains one of the most catastrophic consequences of high-energy trauma, predominantly resulting from motor vehicle collisions, falls from significant heights, and crush injuries [1,2]. Although relatively rare, BAI is associated with a high mortality rate, particularly when left undiagnosed or inadequately treated. Anatomically, the most common injury site is the aortic isthmus, where the mobile aortic arch transitions into the relatively fixed descending thoracic aorta, making it susceptible to shear forces during rapid deceleration [3]. Historically, open surgical repair was the mainstay of treatment, but it carried substantial perioperative risks, especially in patients with multiple concurrent injuries. Over the last two decades, there has been a paradigm shift toward endovascular approaches, primarily thoracic endovascular aortic repair (TEVAR), which offer minimally invasive alternatives with promising outcomes [4]. However, associated polytrauma adds complexity to the clinical picture and raises important questions about the optimal timing, modality, and prioritization of interventions. Management strategies must be tailored to the patient's hemodynamic stability, the severity of concomitant injuries, and institutional resources [5]. Despite advancements in diagnostic imaging and surgical techniques, clinical decision-making remains nuanced, especially in the setting of competing life-threatening injuries [6]. This systematic review aims to evaluate the current evidence surrounding the management of BAI in patients with associated polytrauma, with a particular focus on surgical timing, choice of intervention, and clinical outcomes.

To systematically address the clinical question guiding this review, the PICO model [7] was utilized to define the Population, Intervention, Comparison, and Outcomes. The population (P) under investigation includes patients suffering from BAI in the setting of associated polytrauma, typically resulting from high-impact blunt trauma events. The intervention (I) of interest is endovascular repair, primarily TEVAR, which has emerged as a preferred approach due to its minimally invasive nature and potential to reduce perioperative morbidity. This is compared (C) against open surgical repair, delayed intervention, or non-operative management depending on the study designs and clinical contexts evaluated. The outcomes (O) assessed encompass mortality rates, postoperative complications, timing of intervention, hospital length of stay, and long-term vascular patency. By framing the review within this structured PICO framework, we aim to synthesize existing literature in a manner that is both methodologically rigorous and clinically relevant, facilitating evidence-based recommendations for the management of BAIs in polytrauma patients.

## Review

Materials and methods

Search Strategy

The search strategy for this systematic review was developed by PRISMA (Preferred Reporting Items for Systematic Reviews and Meta-Analyses) guidelines [8] to ensure methodological rigor and transparency. A comprehensive search was conducted across multiple databases, including PubMed, Embase, Scopus, Web of Science, and the Cochrane Library, using a predefined combination of keywords and Boolean operators to capture studies related to blunt thoracic aortic injury (BTAI), polytrauma, and surgical or endovascular management. Search terms included variations of “blunt aortic injury,” “blunt thoracic aortic injury,” “traumatic aortic rupture,” “polytrauma,” “endovascular repair,” and “TEVAR,” combined using AND/OR logic to maximize sensitivity. Studies were screened based on titles and abstracts, followed by a full-text assessment to determine eligibility according to predefined inclusion and exclusion criteria. Only peer-reviewed clinical trials, cohort studies, and relevant narrative reviews published in English were included. Data were extracted independently and organized in structured tables for synthesis. The PRISMA flow diagram was used to document the selection process and illustrate the number of studies identified, screened, assessed for eligibility, and ultimately included in the review.

Eligibility Criteria

The eligibility criteria for this systematic review were defined to ensure the inclusion of high-quality and relevant studies addressing the management of BTAI in the context of polytrauma. Studies were considered eligible if they involved human subjects diagnosed with BTAI resulting from blunt trauma and included data on either endovascular repair (primarily TEVAR) or open surgical intervention. Both prospective and retrospective studies, clinical trials, cohort studies, and narrative reviews were included, provided they reported on outcomes such as mortality, morbidity, technical success, perioperative complications, timing of intervention, or long-term graft durability. The population of interest included adults and adolescents presenting with BTAI, with or without associated multi-system trauma, treated in emergency or trauma center settings.

Exclusion criteria comprised studies focusing exclusively on penetrating aortic injuries, animal or cadaveric models, pediatric patients without clear applicability to adult trauma care, case reports with fewer than five patients, and articles lacking full text or published in languages other than English. Additionally, studies not reporting primary outcomes relevant to intervention efficacy, safety, or decision-making processes were excluded. This approach ensured that the final selection of studies provided meaningful insights into real-world treatment strategies and outcomes for patients with BTAI and associated polytrauma.

Data Extraction

Data extraction was performed systematically using a standardized form designed to capture all relevant variables from the included studies. Key information extracted included author names, year of publication, study design, sample size, population characteristics, type of intervention (e.g., TEVAR or open surgical repair), comparison groups if present, key outcomes measured (such as mortality, morbidity, technical success, complications, reintervention rates, and long-term follow-up findings), timing of intervention, and main conclusions. Each study was reviewed independently, and extracted data were cross-verified to ensure accuracy and consistency. Where applicable, additional details such as injury grading, polytrauma severity, and imaging or procedural techniques were also recorded to enable comprehensive synthesis. Discrepancies in data extraction were resolved through consensus discussion. This structured approach facilitated the qualitative comparison of findings across heterogeneous studies and supported a robust narrative synthesis in alignment with PRISMA guidelines.

Data Analysis and Synthesis

Data analysis and synthesis for this systematic review were conducted using a qualitative, narrative approach due to the heterogeneity of study designs, populations, and outcome measures across the included literature. Rather than performing a meta-analysis, which was limited by the absence of standardized effect sizes and control groups in many studies, findings were synthesized descriptively to highlight trends, patterns, and consistencies in clinical outcomes and management strategies. Studies were grouped based on the type of intervention, timing of treatment, and presence of associated polytrauma, allowing for a thematic comparison of results. The effectiveness and safety of TEVAR were consistently evaluated across studies and compared with outcomes from open surgical repair where applicable. Special attention was given to the impact of injury severity, the timing of intervention (early vs. delayed), and technological advancements influencing procedural success. The synthesis aimed to contextualize evidence within the broader scope of trauma care, offering meaningful clinical insights while identifying gaps in current knowledge and areas requiring further research.

Results

Study Selection Process

**Figure 1:**
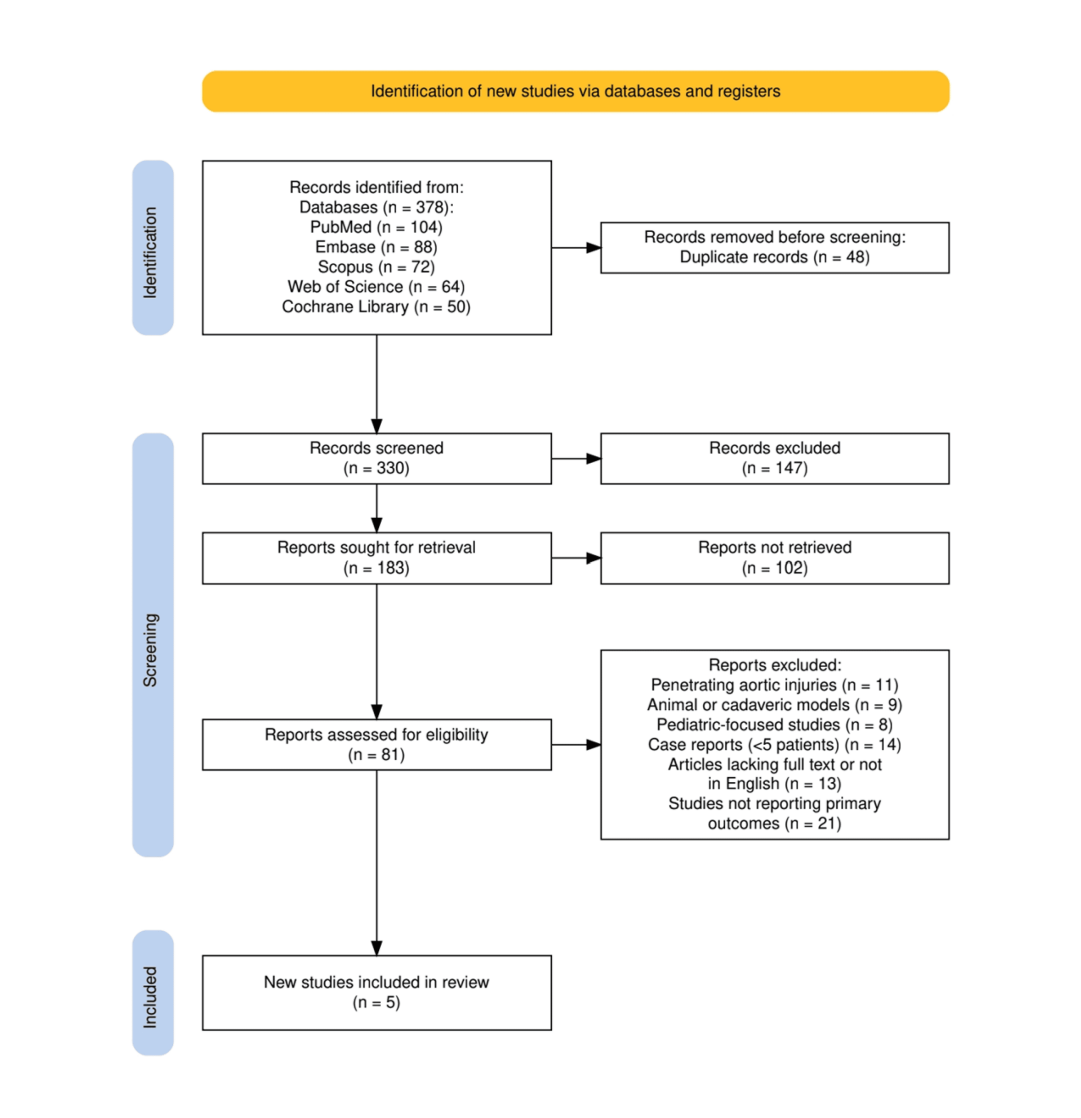
The PRISMA flowchart represents the study selection process. PRISMA, Preferred Reporting Items for Systematic Reviews and Meta-Analyses

The study selection process followed the PRISMA (Preferred Reporting Items for Systematic Reviews and Meta-Analyses) guidelines and is illustrated in Figure 1. A total of 378 records were initially identified through searches across five major databases: PubMed (104), Embase (88), Scopus (72), Web of Science (64), and the Cochrane Library (50). After removing 48 duplicate records, 330 unique studies were screened by title and abstract, resulting in the exclusion of 147 studies that were irrelevant. Of the 183 full-text reports sought for retrieval, 102 could not be retrieved, and the remaining 81 reports were assessed for eligibility. Based on predefined exclusion criteria, 76 reports were excluded for reasons including a focus on penetrating aortic injuries, animal or cadaveric models, pediatric populations, case reports with fewer than five patients, articles lacking full text or not in English, and studies not reporting relevant primary outcomes. Ultimately, five studies met all inclusion criteria and were included in the final systematic review.

Characteristics of the Selected Studies

The characteristics of the studies included in this systematic review are summarized in Table 1 and reflect a diverse range of study designs, populations, and clinical approaches to managing BTAI. Among the five selected studies, three were clinical trials, two prospective and one retrospective, while one was a multicenter retrospective cohort study and another a narrative review. Sample sizes varied significantly, from nine to 209 patients, with populations consisting primarily of adults presenting with BTAI, many in the context of polytrauma. Endovascular repair, particularly TEVAR, was the predominant intervention evaluated across the studies, with open surgical repair either used as a comparator or excluded altogether. Outcomes measured included mortality, morbidity, technical success, complications, and long-term graft durability, with most studies emphasizing the role of intervention timing in relation to injury severity and patient stability. While some studies favored delayed TEVAR in stable patients, others demonstrated the safety and efficacy of acute intervention. Overall, the included studies provided a comprehensive perspective on evolving clinical strategies, supporting the growing consensus around TEVAR as the preferred approach in contemporary trauma care.

**Table 1:** The characteristics of the studies included in this systematic review. BTAI, blunt thoracic aortic injury; TEVAR, thoracic endovascular aortic repair; TAI, traumatic aortic injury; ISS, injury severity score

Author(s) & Year	Study Design	Sample Size	Population Details	Intervention (I)	Comparison (C)	Key Outcomes Measured	Timing of Intervention	Main Findings
Mazzaccaro et al., 2023 [9]	Narrative review/clinical update	Not specified	Patients with BTAI	Endovascular repair (delayed if stable)	Open surgical repair	Mortality, morbidity, need for long-term follow-up	Delayed intervention preferred in stable patients	Endovascular repair associated with lower perioperative mortality and morbidity; delayed repair favored when feasible; injury grade and polytrauma influence management decisions
Boutin et al., 2023 [10]	Multicenter retrospective cohort	209 patients	Patients with BTAI from 10 French trauma centers; mean age 43; 80% male	TEVAR (92% of treated)	Open surgery (10%), some untreated	Prevalence, time to diagnosis, mortality by grade, complications	85% treated within 24 hrs	BTAI was rare (1%) but occurred in severe trauma. Mortality increased with injury severity. TEVAR preferred (90%+ of interventions), with an 18.3% complication rate
Liese-Landolt et al., 2023 [11]	Retrospective cohort study	19 patients	Acute TAI with severe polytrauma treated at a level-1 trauma center (Zurich, 2012–2022)	TEVAR	None (no open repair group)	Technical success, perioperative complications, reintervention, long-term durability	Acute TEVAR; median follow-up 29 months	95% technical success, no intra-op deaths, 5.3% reintervention rate; sustained long-term graft integrity
Starnes et al., 2015 [12]	Prospective, multicenter clinical trial	50 patients	Grade II-IV BTAI at 17 U.S. sites; mean age 43; mean ISS 31±14	TEVAR using Zenith Alpha low-profile graft	No control group	30-day mortality, technical success, complications (stroke, endoleak), blood loss	Acute setting; follow-up initiated post-procedure	100% technical success; 30-day mortality 2% (non-device-related); no paraplegia or rupture; percutaneous access in 44%
Rousseau et al., 1999 [13]	Prospective clinical trial	9 patients	Subacute (n=5) or chronic (n=4) post-traumatic aortic isthmus ruptures; mean age 37; mostly male	Delayed endovascular stent grafting	None	Technical success, complications, pseudoaneurysm exclusion	Delayed (1-8 months; mean 5.4 months)	100% technical success; complications in 2 patients managed successfully; sac thrombosis achieved in all; viable option even in delayed settings

Quality Assessment

The quality assessment of the included studies, as detailed in Table 2, revealed a spectrum of methodological robustness across different study designs. The narrative review was rated as moderate in quality using the Scale for the Assessment of Narrative Review Articles (SANRA) tool, providing relevant clinical insights but lacking a structured search strategy and synthesis. The retrospective cohort studies were evaluated using the Newcastle-Ottawa Scale (NOS), with scores ranging from moderate to moderately low due to limitations such as lack of control groups, potential confounding, and retrospective data collection. The prospective multicenter clinical trial demonstrated good methodological quality when assessed with the NIH Quality Assessment Tool for before-after studies without control groups, owing to its clear objectives, consistent outcome reporting, and well-defined follow-up, despite the absence of a comparator arm. The prospective case series was rated fair to moderate, showing technical strength but limited by a small sample size and brief follow-up. Collectively, while the included studies support the effectiveness of endovascular approaches, their methodological limitations highlight the need for more rigorous prospective research with standardized outcome measures and control groups.

**Table 2:** The quality assessment of the included studies. SANRA, Scale for the Assessment of Narrative Review Articles; NOS, Newcastle-Ottawa Scale; NIH, National Institutes of Health

Author(s) & Year	Study Design	Tool Used	Criteria Assessed	Quality Rating	Comments
Mazzaccaro et al., 2023 [9]	Narrative review	SANRA	Justification of the article, literature search description, referencing, scientific reasoning	Moderate	Provides good background and clinical insight but lacks systematic search methods and structured synthesis
Boutin et al., 2023 [10]	Retrospective cohort	NOS	Selection, comparability, outcome	6/9 - moderate	Good patient selection and outcome assessment, but potential for confounding and lack of control group
Liese-Landolt et al., 2023 [11]	Retrospective cohort	NOS	Selection, comparability, outcome	5/9 - moderate to low	High technical quality and long follow-up, but single-center, retrospective, no comparison group
Starnes et al., 2015 [12]	Prospective multicenter clinical trial	NIH quality assessment tool for before-after (pre-post) studies with no control group	Clear objective, eligibility criteria, outcome measures, follow-up	Good	Strong design with prospective data, standardized outcome reporting, but lacks comparator arm
Rousseau et al., 1999 [13]	Prospective clinical trial	NIH quality assessment tool for case series	Objective, intervention clarity, outcome measures, follow-up, analysis	Fair to moderate	Early evidence, technically strong, but small sample size, no control, and limited follow-up duration

Discussion

The studies included in this systematic review offer a diverse perspective on the evolving management of BTAI, particularly in the context of polytrauma. Mazzaccaro et al. [9], through a narrative review, emphasized the clinical preference for endovascular repair, especially delayed intervention in hemodynamically stable patients, due to its association with lower perioperative mortality and morbidity compared to open surgical repair. Boutin et al. [10] presented a large multicenter retrospective cohort of 209 patients, revealing that although BTAI is rare (1% prevalence), it typically occurs in severely injured trauma patients, with TEVAR accounting for over 90% of treatments and a complication rate of 18.3%. Liese-Landolt et al. [11] contributed data from a single-center cohort involving 19 patients with acute TAI and severe polytrauma, demonstrating excellent technical success (95%) and long-term graft durability with a low reintervention rate of 5.3%, further supporting TEVARs' reliability in complex trauma settings. Starnes et al. [12], through a prospective multicenter trial using the Zenith Alpha low-profile device, reported 100% technical success and a low 30-day mortality rate (2%) in 50 patients, noting high feasibility for percutaneous access and favorable short-term outcomes. Finally, Rousseau et al. [13] evaluated delayed endovascular treatment in nine patients with subacute or chronic traumatic aortic ruptures, achieving complete pseudoaneurysm exclusion in all cases, despite being limited by sample size and technological constraints of the time. Collectively, these findings affirm the central role of TEVAR in contemporary BTAI management, highlight its adaptability to both acute and delayed scenarios, and underline the importance of tailoring intervention timing to injury severity and patient stability.

The findings of this systematic review align closely with the broader body of literature supporting TEVAR as the preferred modality for managing BTAIs [14]. Previous meta-analyses and clinical guidelines have consistently shown that TEVAR offers superior outcomes in terms of reduced perioperative morbidity and mortality compared to open surgical repair, especially in polytrauma patients [15]. The high technical success rates, as demonstrated across multiple studies in this review, echo similar results in earlier trials and registries such as the RESCUE and American Society for Vascular Surgery reports [16]. Furthermore, the review confirms observations from the existing literature that the severity of aortic injury and associated trauma significantly influence both prognosis and the timing of intervention. Despite some variability in sample size and study design, the consensus remains strong across global data that early recognition and appropriately timed TEVAR yield the most favorable outcomes in BTAI patients. The clinical implications of this review are highly relevant for trauma teams, vascular surgeons, and emergency physicians managing critically injured patients. The collective evidence supports TEVAR as the first-line intervention, particularly in hemodynamically stable patients, due to its minimally invasive nature, rapid deployment, and lower risk of complications such as paraplegia, stroke, and aortic rupture [17]. The studies also highlight the feasibility of delayed intervention in stable polytrauma patients, allowing time to address more urgent injuries without compromising aortic repair outcomes. Moreover, the review underscores the importance of integrating long-term surveillance into clinical protocols, especially in younger patients who are more likely to require follow-up due to the implications of radiation exposure and device durability. These findings may inform future revisions of trauma management guidelines and support the wider adoption of TEVAR in both high-resource and evolving trauma care settings [11].

Management of BTAI in polytrauma patients requires a multidisciplinary approach grounded in rapid diagnostic evaluation and prioritization of life-threatening conditions. This review illustrates that decision-making must be individualized, taking into account not only the grade of aortic injury but also the patient’s overall physiologic status, coexisting injuries, and institutional capabilities [18]. The preference for delayed endovascular intervention in stable patients, as highlighted across several studies, reflects a shift in surgical philosophy from emergent open repair to staged, minimally invasive strategies that accommodate the complexity of multiple injuries [19]. The choice between early versus delayed TEVAR, or even conservative management in select low-grade injuries, should be guided by dynamic risk-benefit assessments, resource availability, and evolving clinical parameters [20]. Ultimately, these findings advocate for the establishment of standardized treatment pathways and trauma protocols that emphasize early diagnosis, risk stratification, and the timely application of endovascular technologies to optimize patient outcomes.

The timing of intervention plays a pivotal role in the management of BTAI, especially in patients presenting with associated polytrauma. Several studies in this review support the strategy of delayed intervention in hemodynamically stable patients, allowing clinicians to stabilize other life-threatening injuries before addressing the aortic lesion [21]. This approach not only minimizes perioperative risks but also aligns with current trauma care principles prioritizing damage control. In contrast, immediate repair remains essential for unstable patients or those with high-grade aortic injuries at imminent risk of rupture. The literature reflects that both early and delayed TEVAR can be safe and effective when selected appropriately, emphasizing the need for individualized, context-driven decision-making [22]. Moreover, as demonstrated in multiple cohorts, injury severity and the presence of concomitant trauma significantly influence outcomes, with higher-grade aortic injuries correlating with increased mortality and complications, particularly when diagnosis or intervention is delayed due to resuscitation demands [23].

When comparing TEVAR to open surgical repair, TEVAR consistently demonstrates superior outcomes, particularly in the setting of polytrauma [24]. Across the reviewed studies, TEVAR is associated with higher technical success, reduced blood loss, shorter procedure times, and significantly lower perioperative morbidity and mortality. Open repair, while historically standard, is now largely reserved for cases where endovascular access is not feasible or anatomy is unsuitable [25]. The shift toward TEVAR has been facilitated by significant technological advancements, including the development of low-profile, flexible stent graft systems that accommodate challenging aortic curvatures and allow for percutaneous access even in emergent settings. Devices such as the Zenith Alpha graft exemplify this evolution, enabling safer intervention in a broader patient population [12]. As technology continues to improve, the threshold for offering TEVAR in complex or anatomically difficult cases is expected to lower further, reinforcing its dominance as the preferred treatment modality in modern trauma vascular care.

This systematic review possesses several strengths that enhance its clinical and academic value. It integrates findings from diverse study designs, including multicenter trials, retrospective cohorts, and narrative reviews, offering a broad yet focused perspective on the management of BTAI in polytrauma settings. The inclusion of both recent and foundational studies allows for a comprehensive temporal comparison of evolving practices, particularly highlighting the growing dominance of TEVAR. Furthermore, the structured extraction and quality assessment of studies lend methodological rigor to the synthesis. However, certain limitations must be acknowledged. The heterogeneity of study designs, sample sizes, and outcome measures limits the ability to perform a meta-analysis and may introduce bias in interpreting aggregated results. Additionally, some studies lacked control groups or long-term follow-up, and the review is inherently constrained by the quality and completeness of the available literature. These factors may affect the generalizability of the findings and underscore the need for further high-quality, prospective, comparative studies.

Despite advances in the management of BTAI, several gaps in current knowledge remain. There is limited high-quality comparative data directly evaluating the long-term outcomes of TEVAR versus open surgical repair, particularly in younger patients where concerns about stent durability, radiation exposure, and surveillance burden persist. Additionally, existing literature often lacks standardized reporting on injury grading, polytrauma severity, and intervention timing, which hinders precise stratification of risk and treatment optimization. Most studies are retrospective or observational, and few adequately address the nuances of decision-making in complex polytrauma scenarios. Future research should focus on well-designed, prospective multicenter trials that incorporate uniform diagnostic criteria and outcome measures. Comparative effectiveness studies involving diverse patient populations, including those in low-resource settings, are essential. Furthermore, innovations in imaging, device design, and post-TEVAR follow-up protocols should be investigated to refine clinical pathways and improve both short- and long-term outcomes for BTAI patients.

## Conclusions

The current body of evidence strongly supports TEVAR as the preferred treatment modality for BTAI, particularly in patients presenting with polytrauma. TEVAR consistently demonstrates high technical success rates, lower perioperative morbidity and mortality, and favorable long-term outcomes compared to traditional open surgical repair. The review highlights the importance of individualized intervention timing based on hemodynamic stability and injury severity, as well as the growing role of advanced endovascular technologies in expanding treatment options. While further high-quality comparative studies are needed, the available data affirm TEVAR's role as the cornerstone of modern BTAI management, guiding clinicians toward safer, more effective, and minimally invasive care strategies.
